# Current Practices and Evidence in Caudal Septoplasty: A National Survey and Systematic Review

**DOI:** 10.1093/asjof/ojaf170

**Published:** 2025-12-19

**Authors:** Ahmad Bogari, Khalid Alrasheed, Faisal Alkhunein, Mohammad Aljarba, Mishari Alanezi, Azzam Alotaibi, Abdulaziz Alshehri, Ahmed M Al Arfaj

## Abstract

**Background:**

Caudal septal deviation is a challenging condition that affects nasal function and aesthetics.

**Objectives:**

The aim of the study was to evaluate surgical practices for caudal septoplasty among otolaryngologists in Saudi Arabia and compare them with international approaches.

**Methods:**

A cross-sectional electronic survey was distributed to otolaryngologists across academic, governmental, and private healthcare institutions in Saudi Arabia. A parallel systematic review was conducted following Preferred Reporting Items for Systematic Reviews and Meta-Analyses 2020 guidelines using PubMed, Web of Science, and Scopus. Eligible studies published between 1954 and 2024 described techniques addressing caudal septal deviation with reported outcomes. The study protocol was prospectively registered before the study's start in PROSPERO (CRD42024621207).

**Results:**

Sixty otolaryngologists completed the survey. The most commonly used techniques were swinging door (76.6%), cartilage reshaping (51.7%), and suturing (48.3%). Only 16.7% had facial plastic surgery training. In the systematic review, 559 records were identified with 53 studies meeting the inclusion criteria involving 3564 patients, splinting or grafting (47.2%), and cartilage reshaping (30.2%) predominated. Patient-reported outcome measures (PROMs) were used in 66% of studies, primarily the Nasal Obstruction Symptom Evaluation scale, with scores improving from 70.2 to 16.1 postoperatively.

**Conclusions:**

Otolaryngologists in Saudi Arabia predominantly employ conservative caudal septoplasty techniques, with limited adoption of extracorporeal approaches and PROMs. These findings contrast with broader international practices and highlight the need for enhanced subspecialty training and standardized outcome evaluations.

**Level of Evidence: 5 (Therapeutic):**

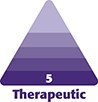

Caudal septal deviation is characterized by the misalignment of the anterior-most portion of the nasal septum, often involving the nasal valve and compromising tip support.^1^ Its etiology is primarily congenital; however, trauma or iatrogenic injury may also contribute to its development.^2^ These deviations result in both functional and aesthetic impairments, including nasal obstruction, lobular deviation, and tip ptosis.^3^ They account for ∼5% to 8% of all nasal septal deviations.^4,5^

Surgical correction of caudal deviations remains challenging because of their anatomical complexity and the risk of destabilizing the nasal tip support.^3^ Techniques, such as the swinging door approach, extracorporeal septoplasty, and graft-based methods, have been developed to address this problem.^6^ However, achieving long-term stability and precise alignment remains challenging.^7^

Evaluation methods include objective tools such as acoustic rhinometry (AR), optical coherence tomography, and paranasal computed tomography (CT).^8^ However, objective findings often poorly correlate with subjective symptoms. Therefore, validated patient-reported outcome measures (PROMs), such as the Nasal Obstruction Symptom Evaluation (NOSE) scale, Rhinoplasty Outcome Evaluation (ROE), and Standardized Cosmesis and Health Nasal Outcomes Survey (SCHNOS), are being increasingly used to assess both functional and aesthetic outcomes.^9-11^ Improper technique selection may lead to structural complications, such as columellar shortening, internal nasal valve collapse, persistent obstruction, and dissatisfaction.^3^

The aim of this study is to assess the international evidence on surgical decision making in the management of caudal septal deviation and to compare Saudi otolaryngologists’ management with that reported in the international literature. This pairing enables direct estimation of evidence–practice gaps and identifies concrete targets for training, standardization, and future controlled evaluation.

## METHODS

### Survey

#### Ethical Approval

This study was approved by the IRB at King Saud University, Riyadh, Saudi Arabia (reference no. 25/0045/IRB), and informed consent was obtained from all participants.

#### Data Collection

A 14-item English language online questionnaire was distributed over 2 months (March-May 2025). The questionnaire was developed by the study authors based on key items from a previous study by Voizard et al, to ensure relevance and comparability.^11^ It was then pilot tested by 3 otolaryngologists at the authors’ institution for clarity and content validity. Distribution was facilitated by departmental heads of major hospitals and the Saudi Otorhinolaryngology Society. Participation was voluntary and anonymous.

#### Data Source Measurements and Statistical Analysis

The survey included questions on surgeon demographics (age, sex, training background, years of experience, and subspecialty), practice setting (university, governmental, or private), and monthly volume of caudal septal deviation cases. The participants were also asked about the surgical techniques used, methods of preoperative and postoperative evaluation, follow-up duration, and reported complications.

Seven predefined surgical technique categories were presented to respondents, each with a clear descriptive label: (1) swinging door technique—techniques that reposition the lowermost part of the caudal septum to the midline or to the contralateral side of the anterior nasal spine; (2) extracorporeal technique—the removal of the deviated septal cartilage with or without the bony septum, followed by extracorporeal reshaping and complete reimplantation; (3) splinting or grafting technique—cartilage splinting and various bony or cartilaginous grafts used to stabilize the nasal septum. Cartilage grafts consist of posterior septal, quadrangular, costal, or conchal cartilage. Bone grafts include bony batten grafts of various origins; (4) suturing technique—all techniques involving permanent trans-cartilaginous retention sutures; (5) cartilage reshaping technique—all methods modifying cartilage structure to correct deviations. This comprises scoring, cross-hatching, wedge resection, cutting and overlapping, and cartilage splitting and repositioning; (6) anterior spine maneuvers—techniques that modify the anterior nasal spine to improve septal alignment and stability. Examples include anterior spine reduction, repositioning, and reshaping; and (7) artificial implants—nonautologous materials used for septal support. Examples include polydioxanone plates and 3-dimentional printed implants designed to stabilize or replace deficient septal segments. Responses were exported to a spreadsheet software and analyzed descriptively using frequencies and percentages.

### Systematic Review

#### Literature Search Strategy

This systematic review was conducted in accordance with the Preferred Reporting Items for Systematic Reviews and Meta-Analyses (PRISMA) 2020 guidelines.^12^ A PRISMA checklist is reported in Supplemental Figures 1 and 2. The study protocol was registered in the National Institute of Health Research Prospective Register of Systematic Reviews (PROSPERO; ID: CRD42024621207) before the initiation of the study.^13^ A comprehensive literature search was conducted using PubMed, Web of Science, and Scopus to identify studies published between 1954 and December 2024. The search string utilized for all databases included (“caudal deviation” OR “caudal septal deviation” OR “anterior deviation” OR “anterior septal deviation”) based on previous work by Voizard et al.^11^

#### Methodology for Selecting Studies

The searched studies had to meet the following inclusion criteria: (1) the study was published in English; (2) the study reported the number of patients for whom outcomes were measured, and the number of controls, if present; (3) the study provided a detailed description of the surgical technique (either septoplasty or septorhinoplasty) aimed to correct caudal septal deviation, offering sufficient detail to understand the steps of the surgery and differentiate it from other techniques; (4) the procedure addressed functional symptoms or cosmetic complaints; (5) the study detailed how functional or cosmetic outcomes were evaluated; and (6) the study was based on an original article, such as a prospective or retrospective cohort study, case–control study, cross-sectional study, randomized or nonrandomized controlled trial, or case series. The exclusion criteria were as follows: procedures addressing noncaudal nasal septal deviations; literature reviews, editorials, correspondences, conference abstracts, descriptions of techniques without outcome evaluation; book chapters; and case reports.

#### Screening and Data Extraction

After removing duplicates, 2 authors (F.A., Mo.A.) independently screened the titles and abstracts using Rayyan (Rayyan Systems Inc., Qatar).^14^ Full texts were then reviewed for eligibility, and disagreements were resolved by consensus or by a third reviewer (K.A.). The full text was reviewed, when the title and abstract did not provide enough information during screening.

Data were independently extracted by 4 authors using a standardized data extraction sheet (F.A., Mo.A., Mi.A., Az.A.). The following data were extracted from each selected study: author, publication date, country, journal, study design, number of patients, surgical technique, postoperative outcome evaluation method, and follow-up (in months). Any discrepancies in the data extraction were resolved through discussion with the fifth author (A.B.).

Surgical techniques were classified into distinct categories using the same 7 definitions provided to the survey participants (see Survey section). Records detailing combinations or sequences of techniques were categorized under multiple classifications, as appropriate.

Postoperative outcome evaluation methods were also classified, including tools such as custom questionnaires (CQ), photographic analysis (PA), clinical examination, endoscopy, rhinomanometry (RM), AR, visual assessment scale, and validated instruments like NOSE, ROE, Sino-Nasal Outcome Test-16 (SNOT-16), and SCHNOS, as well as CT and other measures.

#### Assessment of Quality and Data Analysis

The Methodological Index for Non-Randomized Studies (MINORS) was used to evaluate the quality of the included studies. It is a validated tool for assessing the quality of nonrandomized study designs, including both noncomparative and comparative studies. The index consists of 12 items for noncomparative studies and 8 items for comparative studies. Each item is scored on a scale of 0 to 2, with a maximum score of 16 for noncomparative studies and 24 for comparative studies. For noncomparative studies, a score of ≤8 was considered to be poor quality, 9 to 14 moderate quality, and 15 to 16 good quality. For comparative studies, scores of ≤14, 15 to 22, and 23 to 24 were considered poor, moderate, and good quality, respectively.^15^ Two authors (Mi.A., Az.A.) independently assessed the methodological quality using the MINORS tool. Discrepancies were resolved by consensus, and when needed, by discussion with a third author (K.A.). Qualitative synthesis was performed to summarize the findings. Owing to the significant heterogeneity in the design, techniques, and outcome measures, a meta-analysis was not feasible.

## RESULTS

### Survey

Table 1 represents the demographic characteristics of the survey participants. A total of 60 otolaryngologists completed the survey, of whom 78.3% were male. Most respondents (91.7%) had more than 5 years of professional experience.

**Table 1.** Data on Demographics and Professional Characteristics of Survey Respondents (*n* = 60)

Variable	No. of respondents	Percentage of respondents (%)
Age		
31-40	30	50.0
41-50	22	36.7
51-60	6	10.0
>60	2	3.3
Sex		
Male	47	78.3
Female	13	21.7
Years of practice		
1-5	5	8.3
6-10	24	40.0
11-20	22	36.7
21-30	6	10.0
>30	3	5.0
Performing caudal septoplasty		
Yes	53	88.3
No	7	11.7
Facial plastic and reconstructive surgery fellowship completion		
Yes	10	16.7
No	50	83.7
Training		
Saudi Arabia	45	75.0
North America	4	6.7
Others	11	18.3
Number of caudal deviations seen per month		
0-5	39	65.0
6-10	14	23.3
11-20	6	10.0
>20	1	1.7

**Figure 1.**
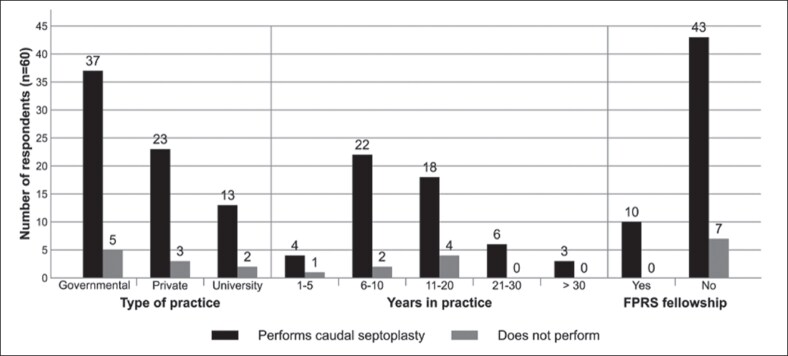
Performance of caudal septoplasty based on practice setting and training. The figure represents survey responses from Saudi otolaryngologists. FPRS, facial plastic and reconstructive surgery.

Most respondents (88.3%) performed caudal septoplasty in their practice, although only 16.7% had completed a facial plastic and reconstructive surgery (FPRS) fellowship (Figure 1). Most completed their training in Saudi Arabia, with North America being the second most common place of training.

Regarding case volume, 65% (*n* = 39) reported seeing 0 to 5 caudal septal deviation cases monthly, and 23.3% (*n* = 14) saw 6 to 10 cases. Among respondents who saw 0 to 5 caudal septal deviation cases in the clinic per month, 10.3% did not perform caudal septoplasty. In comparison, 14.3% of those seeing >5 cases per month did not perform the procedure.

**Figure 2.**
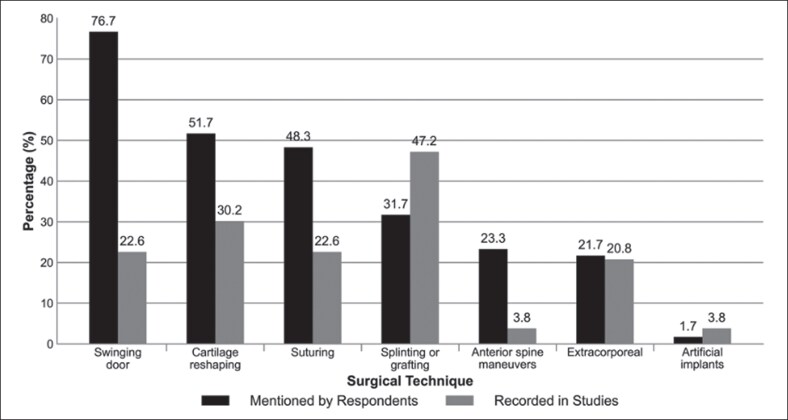
Comparison of caudal septoplasty techniques by Saudi otolaryngologists vs included studies. HPI, history of present illness; PROMs, patient-reported outcome measures.

**Figure 3.**
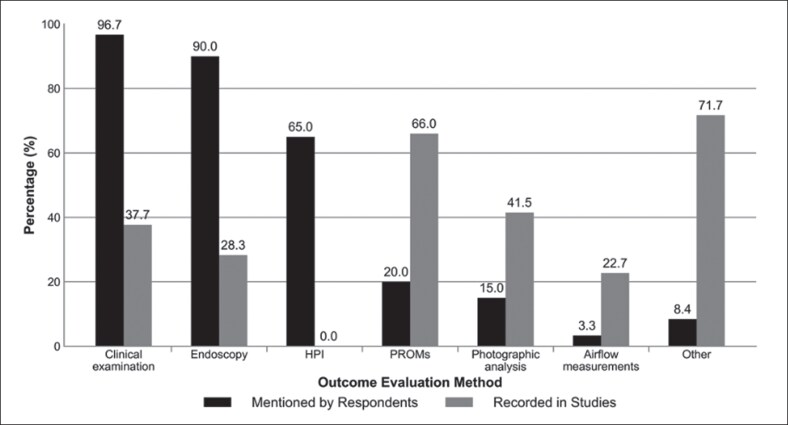
Comparison of outcome evaluation methods by Saudi otolaryngologists vs included studies.

The most frequently reported surgical techniques were the swinging door technique (76.6%), cartilage reshaping (51.7%), and suturing (48.3%; Figure 2). For postoperative evaluation, the most commonly used methods included clinical examination (96.7%), nasal endoscopy (90%), and patient history (65%; Figure 3). Other tools, such as validated PROMs (20.0%) or objective airflow assessments (3.3%), were rarely employed.

The follow-up duration varied, with 28.3% conducting evaluations at 12 months and 23.3% at 6 months, with an average follow-up period of 5.8 months. The most frequently reported complications were persistent caudal deviation (83.3%), nasal obstruction (40%), and nasal tip ptosis (16.7%). Less common complications were not consistently encountered.

### Systematic Review

#### Literature Findings and Characteristics of the Included Studies

**Figure 4.**
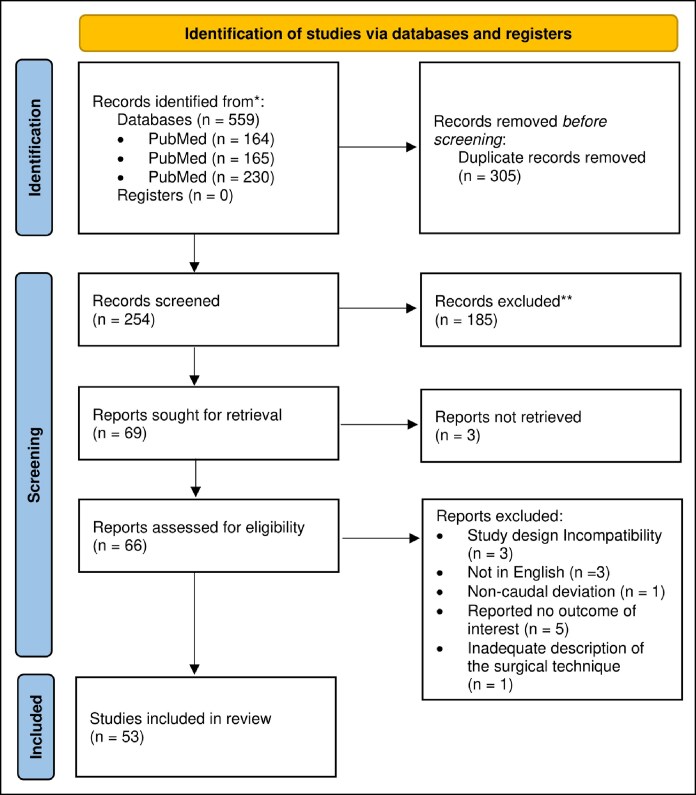
Preferred Reporting Items for Systematic Reviews and Meta-Analyses flow diagram.

A total of 559 studies were identified: 164 from PubMed, 165 from Web of Science, and 230 from Scopus. After removing duplicates, 254 records were retained for screening. Of these, 69 underwent full-text review and 53 met the final inclusion criteria (Figure 4).^5,6,16-66^ All studies were published between 1994 and 2024, including 36 retrospective and 17 prospective studies. Twelve studies each were conducted in the United States and South Korea, followed by 8 in Turkey, and 3 each in Iran and Egypt. Other countries included Japan, Italy, France, Saudi Arabia, and Brazil.

The total number of patients across the studies was 3564, with individual sample sizes ranging from 2 to 703 (Supplemental Table 1). The participants’ ages ranged from 5 to 93 years. Only 8 studies (15%) included a control group.^16,27,33,34,46,47,55,61^

#### Surgical Techniques Used in the Included Studies

The most frequently reported surgical technique was splinting or grafting (47.2%), followed by cartilage reshaping (30.2%). Swinging door and suturing techniques were each reported in 22.6% of the studies. Extracorporeal septoplasty was performed in 20.8% of the studies as either a primary or an adjunctive procedure. Less commonly reported techniques included anterior spine maneuvers and artificial implants (3.8% each; Figure 2).

#### Outcome Evaluation Methods and Follow-up Duration

Postoperative outcomes were most commonly evaluated using validated PROMs, reported in 66% of the studies. The NOSE scale was the most frequently used tool (52.8%), followed by the ROE (7.5%), SCHNOS (3.8%), and SNOT-16 (1.9%).^19,20,29,31,45,46^ Among the 27 studies reporting NOSE scores, the average value improved from 70.2 preoperatively (range, 42.4-90) to 16.1 postoperatively (range, 3.75-32.1; Table 2). PA and CQs were each used in 41.5% of studies. Among those using CQs, only 5 studies disclosed the actual questionnaire content.^18,35,51,55,66^ Four studies used PA as the sole assessment tool.^34,49,50,52^

**Table 2.** Mean Preoperative and Postoperative NOSE Scores (*n* = 27)

Author, year	No. of patients	Surgical technique	Preoperative NOSE score	Postoperative NOSE score	ΔNOSE score (pre–post)
Ghosh, 2024^16^	12	Swinging door	72.5	23.3	49.2
Moon, 2023^17^	20	Suturing, Cartilage reshaping, Artificial implants	68.2	15.0	53.2
İşlek, 2023^19^	77	Swinging door, splinting or grafting	42.4^a^	6.8^a^	35.6
Sabino, 2022^20^	95	Suturing, cartilage reshaping	75.5	11.3	64.2
Chi, 2022^21^	26	Splinting or grafting	75.4	14.0	61.3
Hosokawa, 2021^23^	22	Swinging door, suturing	77.5	5.0	72.5
Aksakal, 2021^24^	35	Suturing	80.2	21.2	59.0
Awan, 2021^27^	28	Extracorporeal, cartilage reshaping	67.4	15.8	51.5
Aksakal, 2020^28^	27	Splinting or grafting, suturing	85.1	22.4	62.7
Demir, 2020^29^	43	Extracorporeal, cartilage reshaping	90.0^a^	10.0^a^	80.0^a^
Yağmur, 2020^30^	26	Extracorporeal	79.0^a^	25.5^a^	53.5^a^
Patel, 2020^31^	58	Extracorporeal	65.3	17.9	47.4
Seo, 2020^32^	67	Suturing	52.9	5.8	47.1
Kim, 2019^35^	29	Splinting or grafting	62.1	32.1	30.0
Cheon, 2019^36^	31	Suturing, cartilage reshaping	43.5	11.0	32.5
Joo, 2019^37^	50	Suturing, cartilage reshaping	67.5^a^	20.0^a^	47.5^a^
Kim, 2018^39^	20	Artificial implants	73.5	3.8	69.8
Ghorbani, 2018^40^	14	Swinging door, cartilage reshaping, splinting or grafting, anterior spine maneuvers	67.5	10.0	57.5
Lee, 2018^41^	22	Splinting or grafting	72.0	8.0	64.0
Kim, 2017^6^	141	Splinting or grafting	70.5	28.7	41.8
Loyo, 2017^42^	71	Extracorporeal	72.3	24.0	48.2
Karadavut, 2016^46^	20	Splinting or grafting	64.0	12.0	52.0
Kayabasoglu, 2015^47^	45	Extracorporeal	85.0	25.0	60.0
Surowitz, 2015^48^	77	Extracorporeal	68.2	21.1	47.1
Garcia, 2011^56^	10	Splinting or grafting	83.5	7.5	76.0
Giacomini, 2010^58^	15	Splinting or grafting; Cartilage reshaping	57.4^a^	23.7^a^	33.7^a^
Most, 2006^60^	12	Extracorporeal, splinting or grafting	76.7	12.9	63.8
Total (pooled)	1093	—	68.8	17.3	51.4

^a^The Nasal Obstruction Symptom Evaluation (NOSE) scores were recalculated as they were not originally reported in accordance with the standard NOSE score calculation method outlined in the respective article.

Clinical examinations were performed in 37.7% of the studies, whereas endoscopy and CT were performed in 28.3% and 5.7% of the studies, respectively. A visual assessment scale was used in 24.5% of the studies. AR and RM were reported in 17% and 5.7% of the studies, respectively (Figure 3). No studies reported a history of the present illness. The mean follow-up duration was ∼11.9 months, most commonly ranging from 6 to 12 months, with an overall range of 1.3 to 112.8 months (Supplemental Table 1).

#### Analyzing Biases, Assessing Quality, and Determining the Level of Evidence

The MINORS tool was used to assess the quality of the included nonrandomized studies (Supplemental Tables 2, 3).^15^ The total scores ranged from 2 to 24, with a mean score of 13.3. The items with the lowest scores were the prospective calculation of the study size (average score: 0.96), prospective collection of data (average score: 1.17), and unbiased assessment of the study endpoint (average score: 1.21). The items with the highest scores were <5% loss to follow-up (average score: 1.75), a clearly stated aim of the study (average score: 1.70), and clearly defined endpoints (average score: 1.68). Overall, 6/53 (11.3%) studies were rated as poor quality, 31/53 (58.5%) as moderate, and 16/53 (30.2%) as good quality. No randomized controlled trials were included in this study. Therefore, the overall level of evidence was limited, and caution should be exercised when interpreting the results of this review. Further high-quality prospective studies are required to provide more robust evidence.

## DISCUSSION

This study evaluated the surgical practices and outcome assessment strategies for caudal septal deviation among otolaryngologists in Saudi Arabia, and compared them with international benchmarks using a systematic review and a previous North American survey.^11^ Given the dual functional and aesthetic impacts of caudal deviation, the alignment of practices with evidence-based standards is essential.^35,67^

Locally, swinging door (76.6%), cartilage reshaping (51.7%), and suturing (48.3%) were the most common procedures. In contrast, North American data showed similar use of swinging door (69.5%) and scoring (45.3%), but significantly higher adoption of extracorporeal septoplasty (46.7% vs 21.7%), potentially reflecting training gaps (Figure 2).^11,68^

This systematic review provides further insights into the variability in surgical practice and global trends. Splinting and grafting techniques appeared most frequently (47.2%), followed by cartilage reshaping (30.2%), whereas other methods such as swinging door, suturing, and extracorporeal septoplasty have also been reported with varying prevalence. Less common techniques, such as anterior spine maneuvers and artificial implants, were infrequent (3.8%). This range reflects the influence of surgeon preference, anatomical challenges, and available expertise on the choice of surgical approach.^6,67,69^

One of the most notable findings across all data sources was the limited use of validated PROMs by Saudi surgeons. Although most relied on physical examinations (96.7%), nasal endoscopy (90%), and patient history (65%), few used tools such as NOSE, ROE, or SCHNOS scales. Similarly, Voizard et al reported that only 26.6% of North American clinicians routinely used PROMs in practice.^11^ Conversely, 66% of the reviewed studies used PROMs, especially the NOSE scale (52.8%), underscoring a clinical research gap.^9,10^ Moreover, objective measures such as AR and RM were underused (17% and 5.7%, respectively) and nearly absent in local practice, possibly because of limited access, cost, or interpretation challenges.^70^

The systematic review showed a substantial improvement in NOSE scores across techniques, with an average preoperative score of 70.2 improving to 16.1 postoperatively. These findings support the effectiveness of multiple surgical approaches in improving patient-reported nasal obstruction. However, only 15% of the studies included a control group and the overall quality of evidence was highly variable, with many studies scoring poorly on prospective design, unbiased endpoint assessment, and statistical power based on the MINORS tool.

In terms of complications, both Saudi and North American surveys identified persistent deviation and nasal obstruction as the most common complications. The North American study noted similar outcomes and further highlighted structural complications, such as tip ptosis and external nasal valve collapse, as consequences of an improper caudal septoplasty technique.^3,11^ Although less frequently mentioned in our study, these complications remain clinically significant, particularly when structural support is compromised.

The findings of this study have important implications for clinical practice in the management of caudal septal deviation, especially because certain techniques are more widely adopted in our region. The widespread use of the swinging door technique by Saudi surgeons reflects its practicality and effectiveness in routine cases.^71^ However, the underutilization of more advanced methods, such as extracorporeal septoplasty, suggests a potential area for further surgical training and specialization, particularly in facial plastic surgery, where Surowitz et al and McGrath et al have reported significant improvements in both functional and aesthetic outcomes using this approach.^48,72^ The discrepancy between clinical assessment methods and the use of validated PROMs highlights a critical gap in standardized outcome measurements. Encouraging the routine implementation of PROMs such as the NOSE, ROE, and SCHNOS scales could enhance patient-centered care and provide a more objective basis for evaluating surgical success.^9,73,74^ Aligning local practices with international standards and evidence-based protocols is crucial for enhancing surgical outcomes and will ultimately support improved functional and aesthetic outcomes while facilitating meaningful comparisons across institutions and regions.^16,75^

This study had several limitations that should be acknowledged when interpreting the findings. First, the survey relied on self-reported data from otolaryngologists in Saudi Arabia, which may have introduced recall bias. Additionally, although efforts were made to reach a broad range of ENT physicians, because the survey was distributed through departmental heads and the national society, the total number of invited surgeons could not be ascertained. Therefore, a formal response rate could not be calculated, introducing a potential for nonresponse bias. According to the latest Saudi Ministry of Health statistical report, Saudi Arabia currently has 832 registered board-certified consultant otolaryngologists.^76^ Given this benchmark, the 60 respondents in our study represent 7.2% of this population, which may not fully represent the diversity of surgical practices across all regions and healthcare settings in the country. The relatively low response rate of surgeons with formal FPRS fellowship training (16.7%) also limited the generalizability of the findings related to advanced surgical techniques.

Another limitation was the heterogeneity of the studies included in the systematic review. The variations in study designs, patient populations, surgical techniques, and outcome evaluation methods precluded a quantitative meta-analysis. Most of the included studies were retrospective with a wide range of methodological qualities, as indicated by their varying scores on the MINORS assessment. Furthermore, the wide variation in follow-up in both literature and clinical practice complicates the evaluation of long-term efficacy and patient satisfaction.

Given these limitations, future efforts should prioritize well-designed randomized controlled trials or, where infeasible, prospective cohort studies that directly compare conservative techniques (eg, swinging door) with extracorporeal septoplasty. These studies should employ standardized descriptions of surgical techniques and use of validated PROMs including NOSE scale, with a minimum follow-up of 12 months, to allow for meaningful data synthesis. Additionally, residency and fellowship training programs should be reviewed and potentially updated to ensure adequate exposure to complex techniques, such as extracorporeal septoplasty. Finally, encouraging international collaborations and establishing national surgical registries could help to standardize practice, improve the accuracy of data collection, and provide valuable insights that enhance both patient care and the effectiveness of surgical interventions for caudal septal deviation.

## CONCLUSIONS

Caudal septal deviation remains a complex clinical entity that affects both nasal function and external contour. This study integrated a national survey with a systematic review to provide a comprehensive overview of the current surgical practices and assessment strategies. Among Saudi surgeons, swinging door and cartilage reshaping techniques are most commonly used, with limited integration of validated PROMs. This systematic review revealed a greater emphasis on objective tools and PROMs in published studies, underscoring the gap between clinical implementation and research protocols. These findings further demonstrate the need to expand the use of standardized assessment tools in daily practice. Although locally underutilized, extracorporeal septoplasty is well supported by international evidence and may offer satisfactory outcomes in appropriately selected cases. However, this evidence is drawn primarily from nonrandomized studies of variable quality, as indicated in the MINORS assessment, with few controlled comparisons and no randomized clinical trials. Future improvements in clinical outcomes will depend on enhanced surgical training, routine use of validated evaluation instruments, and the establishment of national outcome registries to support data-driven practice refinement.

## Supplemental Material

This article contains supplemental material located online at https://doi.org/10.1093/asjof/ojaf170.

## Supplementary Material

ojaf170_Supplementary_Data
