# Supplementary material for: Current Practices and Evidence in Caudal Septoplasty: A National Survey and Systematic Review
Source: Aesthet Surg J Open Forum. 2025 Dec 19;8:ojaf170. doi: 10.1093/asjof/ojaf170 (PMC12862218; doi:10.1093/asjof/ojaf170)
Supplement: ojaf170_Supplementary_Data [file ojaf170_supplementary_data.zip › Supplemental Figure Legend.docx]

**Supplemental Figure Legend**

**Supplemental Figure 1.** PRISMA Checklist.

**Supplemental Figure 2.** PRISMA Checklist.
