# Supplementary material for: Current Practices and Evidence in Caudal Septoplasty: A National Survey and Systematic Review
Source: Aesthet Surg J Open Forum. 2025 Dec 19;8:ojaf170. doi: 10.1093/asjof/ojaf170 (PMC12862218; doi:10.1093/asjof/ojaf170)
Supplement: ojaf170_Supplementary_Data [file ojaf170_supplementary_data.zip › Supplemental Table 1.docx]

**Supplemental Table 1.** Overview of the Included Studies

| Author, year | Country | Journal | Study design | Number of patients | Surgical Technique | Outcome evaluation methods | Follow up in months |
| --- | --- | --- | --- | --- | --- | --- | --- |
| Ghosh, 2024^16^ | India | Indian Journal of Otolaryngology and Head & Neck Surgery | P^C^ | 12 | Swinging Door | NOSE; CE; CQ | 6 |
| Moon, 2023^17^ | South Korea | Journal of Otolaryngology - Head & Neck Surgery | R | 20 | Suturing; Cartilage reshaping; Artificial implants | CE; E; CQ; NOSE; VAS | 112.8^*^ |
| Hosnani, 2023^18^ | Iran | International Archives of Otorhinolaryngology | R | 50 | Splinting or Grafting | PA; CQ | 12 |
| İşlek, 2023^19^ | Turkey | European Journal of Plastic Surgery | R | 77 | Swinging Door; Splinting or Grafting | NOSE, ROE | 12 |
| Sabino, 2022^20^ | Italy | European Archives of Oto-rhinolaryngology | P | 95 | Suturing; cartilage reshaping | NOSE, ROE | 12 |
| Chi , 2022^21^ | Taiwan | Healthcare | R | 26 | Splinting or Grafting | NOSE; VAS | 6 |
| Gelidan, 2021^22^ | Saudi Arabia | Plastic and Reconstructive Surgery -Global Open | R | 6 | Splinting or Grafting | PA; CQ | 6 |
| Hosokawa, 2021^23^ | Japan | Journal of Otolaryngology - Head & Neck Surgery | R | 22 | Swinging Door; Suturing | NOSE; CT; CQ | 12^*^ |
| Aksakal, 2021^24^ | Turkey | The Journal of Craniofacial Surgery | R | 35 | Suturing | NOSE; E; CQ | 11.3 ^*^ |
| Nofal, 2021^25^ | Egypt | OTO Open | P | 40 | Suturing | CE; E | 18 |
| Sabry, 2021^26^ | Egypt | The Egyptian Journal of Otolaryngology | P | 30 | Cartilage reshaping | NOSE, VAS | 3 |
| Awan, 2021^27^ | Pakistan | Journal of the College of Physicians and Surgeons Pakistan | R^C^ | 28 | Extracorporeal;Cartilage reshaping | NOSE; VAS | 6 |
| Aksakal, 2020^28^ | Turkey | Turkish Archives of Otorhinolaryngology | R | 27 | Splinting or Grafting; Cartilage reshaping | NOSE; CQ; CE; E | 13.9^*^ |
| Demir, 2020^29^ | Turkey | International journal Auris Nasus Larynx | P | 43 | Extracorporeal;Cartilage reshaping | NOSE; SCHNOS; PA | 6 |
| Yağmur, 2020^30^ | Turkey | Aesthetic Plastic Surgery | R | 26 | Extracorporeal | PA; NOSE; VAS | 17.96^*^ |
| Patel, 2020^31^ | USA | The Laryngoscope | R | 58 | Extracorporeal | NOSE; SCHNOS | 5.23^*^ |
| Seo, 2020^32^ | South Korea | The Laryngoscope | P | 67 | Suturing | VAS; NOSE; E; AR | 6 |
| Lip Ng, 2019^33^ | Canada | Aesthetic Plastic Surgery | R^C^ | 333 | Anterior Spine Maneuvers | CE, CQ | N/A |
| Sazgar, 2019^34^ | Iran | Brazilian Journal of Otorhinolaryngology | R^C^ | 457 | Splinting or Grafting | PA | 17.4^*^ |
| Kim, 2019^35^ | South Korea | Annals of Otology, Rhinology & Laryngology | R | 29 | Splinting or Grafting | NOSE; E; CQ | 1.3^*^ |
| Cheon, 2019^36^ | South Korea | American Journal of Rhinology & Allergy | R | 31 | Suturing; cartilage reshaping | NOSE, PA; AR | 3 |
| Joo, 2019^37^ | South Korea | International Forum of Allergy & Rhinology | R | 50 | Suturing; Cartilage reshaping | NOSE; E | 13^*^ |
| Iimura, 2019^38^ | Japan | Auris Nasus Larynx Head Neck | P | 16 | Suturing; Cartilage reshaping | E; CQ; CT | 12 |
| Kim, 2018^39^ | South Korea | JAMA Otolaryngology–Head &Neck Surgery | P | 20 | Artificial implants | NOSE; AR; VAS; CT | 3 |
| Ghorbani, 2018^40^ | Iran | Indian Journal of Otolaryngology and Head & Neck Surgery | R | 14 | Swinging Door; Cartilage reshaping; Splinting or Grafting; Anterior Spine maneuvers | NOSE; PA | 6 |
| Chan Lee 2018^41^ | Taiwan | The journal Clinical Otolaryngology | R | 22 | Splinting or Grafting | NOSE; CE, RM | 3 |
| Kim, 2017^6^ | South Korea | The Journal of the American Medical Association Facial Plastic Surgery. | R | 141 | Splinting or Grafting | E; CQ; NOSE | 7.6^*^ |
| Loyo, 2017^42^ | USA | The Journal of the American Medical Association Facial Plastic Surgery | R | 71 | Extracorporeal | NOSE; PA | 12 |
| Indeyeva, 2017^43^ | USA | International Journal of Oral & Maxillofacial Surgery | R | 148 | Suturing; Cartilage reshaping | CE | 6 |
| Aboul Wafa, 2017^44^ | Egypt | Plastic and Reconstructive Surgery - Global Open | P | 18 | Swinging Door; Splinting or Grafting | CQ; PA | N/A |
| Yaniv, 2016^45^ | Israel | International Forum of Allergy & Rhinology | P | 63 | Extracorporeal; Cartilage reshaping | CE; E; ROE; SNOT-16 | 26^*^ |
| Karadavut, 2016^46^ | Turkey | Brazilian Journal of Otorhinolaryngology | R^C^ | 20 | Splinting or Grafting | CE; AR; NOSE; ROE | 8 |
| Kayabasoglu, 2015^47^ | Turkey | Ear, Nose & Throat Journal | R^C^ | 45 | Extracorporeal | CE; NOSE; PA;E; VAS | 15.4^*^ |
| Surowitz, 2015^48^ | USA | Otolaryngology–Head and Neck Surgery (OTO Journal) | R | 77 | Extracorporeal | NOSE; VAS | 4.7^*^ |
| Yi, 2014^49^ | South Korea | Annals of Otology, Rhinology & Laryngology | R | 52 | Suturing; Splinting or Grafting; Cartilage reshaping | PA | N/A |
| Constantine, 2014^50^ | USA | Plastic and Reconstructive Surgery | P | 2 | Swinging Door; Splinting or Grafting | PA | 2 |
| Chung, 2013^51^ | South Korea | The Laryngoscope | P | 39 | Swinging Door; Splinting or Grafting | CE; E; AR; VAS; CQ | 6.9^*^ |
| Akduman, 2013^52^ | Turkey | European Archives of Oto-rhino-laryngology | R | 36 | Swinging Door | PA | 10^*^ |
| Lee, 2013^53^ | USA | JAMA Facial Plastic Surgery | R | 66 | Splinting or Grafting | CE; CQ; PA | 8^*^ |
| Shin, 2011^54^ | South Korea | Clinical Otolaryngology | R | 40 | Swinging Door; Suturing | AR; VAS | 3 |
| Kim, 2011^55^ | South Korea | American Journal of Rhinology and Allergy | R^C^ | 56 | Splinting or Grafting; Cartilage reshaping | VAS; CQ | 12.2^*^ |
| Garcia, 2011^56^ | Brazil | Brazilian Journal of Otorhinolaryngology | P | 10 | Splinting or Grafting | NOSE; PA; AR; CE. | 2 |
| Koch, 2011^57^ | USA | Archives of Facial Plastic Surgery | R | 8 | Extracorporeal; Splinting or Grafting | CQ, PA | 12 |
| Giacomini, 2010^58^ | Italy | Annals of Plastic surgery | P | 15 | Splinting or Grafting; Cartilage reshaping | CE; RM; NOSE; PA | 6 |
| Jang, 2009^59^ | South Korea | JAMA Otolaryngology - Head & Neck Surgery) | R | 45 | Splinting or Grafting | E; VAS | 15^*^ |
| Most, 2006^60^ | USA | Archives of Facial Plastic Surgery | P | 12 | Extracorporeal; Splinting or Grafting | NOSE; PA | 5.4^*^ |
| Sedwick, 2005^5^ | USA | Archives of Facial Plastic Surgery | R | 62 | Swinging Door | PA; CQ | 6 |
| Calderón-Cuéllar, 2003^61^ | Mexico | Plastic and Reconstructive Surgery | R^C^ | 25 | Splinting or Grafting; Cartilage reshaping | CE; RM; CQ | 6 |
| Dyer,  2000^62^ | USA | Arch Otolaryngol Head Neck Surg | P | 36 | Swinging Door; Splinting or Grafting | CE; CQ; PA | 59.8^*^ |
| Murrell, 2000^63^ | USA | American Journal of Otolaryngology | R | 10 | Extracorporeal | CE; PA | 12 |
| Kamami, 2000^64^ | France | Otolaryngology– Head and Neck Surgery | R | 703 | Other | CE; E; AR; CQ | 11.8^*^ |
| Kamami, 1997^65^ | France | Journal of Clinical Laser Medicine & Surgery | R | 120 | Other | CE; E; AR; CQ | 6^*^ |
| Metzinger, 1994^66^ | USA | Arch Otolaryngol Head Neck Surg | P | 10 | Swinging Door; Splinting or Grafting | CQ; PA; CE | 12 |

- ^*^Mean Follow up values
- ^C^Has a control
- USA: United States of America; R: retrospective; P: prospective
- CQ: Custom questionnaire, PA: Photographic analysis, CE: Clinical examination, E: Endoscopy, HPI: History of present illness, RM: Rhinomanometry, AR: Acoustic rhinometry, VAS: Visual assessment scale, NOSE: Nasal Obstruction Symptom Evaluation, ROE: rhinoplasty outcome evaluation, SNOT-16: SinoNasal Outcome Test-16, SCHNOS: Standardized Cosmesis and Health Nasal Outcomes Survey, CT: Computed tomography
