# Supplementary material for: Current Practices and Evidence in Caudal Septoplasty: A National Survey and Systematic Review
Source: Aesthet Surg J Open Forum. 2025 Dec 19;8:ojaf170. doi: 10.1093/asjof/ojaf170 (PMC12862218; doi:10.1093/asjof/ojaf170)
Supplement: ojaf170_Supplementary_Data [file ojaf170_supplementary_data.zip › Supplemental Table 2.docx]

**Supplemental Table 2.** MINORS Assessment Tool for Non-randomized Non-comparative Studies (n = 45)

| Item | A clearly stated aim | Inclusion of consecutive patients | Prospective collection of data | Endpoints appropriate to the aim of the study | Unbiased assessment of the study endpoint | Follow-up period appropriate to the aim of the study | Loss to follow-up less than 5% | Prospective calculation of the study size | Total Score |
| --- | --- | --- | --- | --- | --- | --- | --- | --- | --- |
| Moon, 2023^17^ | 2 | 1 | 2 | 2 | 2 | 2 | 2 | 1 | 14 |
| Hosnani, 2023^18^ | 2 | 1 | 2 | 2 | 1 | 2 | 2 | 2 | 14 |
| İşlek, 2023^19^ | 2 | 1 | 2 | 2 | 2 | 2 | 2 | 2 | 15 |
| Sabino, 2022^20^ | 2 | 2 | 2 | 2 | 1 | 2 | 1 | 1 | 13 |
| Chi , 2022^21^ | 2 | 1 | 0 | 2 | 2 | 2 | 2 | 1 | 12 |
| Gelidan, 2021^22^ | 2 | 1 | 0 | 2 | 0 | 2 | 2 | 1 | 10 |
| Hosokawa, 2021^23^ | 2 | 1 | 1 | 2 | 2 | 2 | 2 | 1 | 13 |
| Aksakal, 2021^24^ | 2 | 2 | 2 | 2 | 1 | 2 | 2 | 1 | 14 |
| Nofal, 2021^25^ | 2 | 2 | 0 | 1 | 0 | 2 | 2 | 1 | 10 |
| Sabry, 2021^26^ | 2 | 2 | 2 | 2 | 2 | 2 | 2 | 1 | 15 |
| Aksakal, 2020^28^ | 2 | 2 | 2 | 2 | 2 | 2 | 2 | 0 | 14 |
| Demir, 2020^29^ | 2 | 1 | 2 | 2 | 2 | 2 | 2 | 2 | 15 |
| Yağmur, 2020^30^ | 2 | 1 | 0 | 2 | 2 | 2 | 2 | 1 | 12 |
| Patel, 2020^31^ | 2 | 2 | 2 | 2 | 2 | 2 | 2 | 2 | 16 |
| Seo, 2020^32^ | 2 | 2 | 2 | 2 | 2 | 2 | 2 | 1 | 15 |
| Kim, 2019^35^ | 2 | 1 | 1 | 2 | 2 | 1 | 1 | 1 | 11 |
| Cheon, 2019^36^ | 1 | 2 | 2 | 1 | 0 | 2 | 2 | 1 | 11 |
| Joo, 2019^37^ | 0 | 2 | 2 | 0 | 2 | 2 | 2 | 1 | 11 |
| Iimura, 2019^38^ | 2 | 0 | 2 | 2 | 1 | 2 | 2 | 1 | 12 |
| Kim, 2018 ^39^ | 2 | 2 | 1 | 2 | 2 | 2 | 2 | 2 | 15 |
| Ghorbani, 2018^40^ | 2 | 2 | 2 | 2 | 2 | 2 | 2 | 1 | 15 |
| Chan Lee 2018^41^ | 0 | 2 | 2 | 1 | 2 | 2 | 2 | 2 | 13 |
| Kim, 2017^6^ | 2 | 2 | 2 | 2 | 2 | 2 | 2 | 1 | 15 |
| Loyo, 2017 ^42^ | 2 | 1 | 2 | 2 | 2 | 2 | 2 | 2 | 15 |
| Indeyeva, 2017^43^ | 0 | 2 | 0 | 0 | 1 | 2 | 2 | 1 | 8 |
| Aboul Wafa, 2017 ^44^ | 1 | 1 | 0 | 0 | 0 | 0 | 0 | 0 | 2 |
| Yaniv, 2016^45^ | 2 | 2 | 2 | 2 | 2 | 2 | 2 | 1 | 15 |
| Surowitz, 2015^48^ | 2 | 2 | 1 | 2 | 2 | 1 | 2 | 1 | 13 |
| Yi, 2014^49^ | 2 | 1 | 1 | 2 | 1 | 1 | 1 | 2 | 11 |
| Constantine, 2014^50^ | 0 | 0 | 0 | 0 | 0 | 1 | 2 | 0 | 3 |
| Chung, 2013^51^ | 2 | 2 | 2 | 2 | 2 | 2 | 2 | 2 | 16 |
| Akduman, 2013^52^ | 2 | 1 | 1 | 2 | 1 | 2 | 2 | 1 | 12 |
| Lee, 2013^53^ | 2 | 1 | 0 | 2 | 1 | 1 | 2 | 1 | 10 |
| Shin, 2011^54^ | 1 | 0 | 0 | 0 | 0 | 2 | 2 | 0 | 5 |
| Garcia, 2011^56^ | 2 | 1 | 2 | 2 | 2 | 1 | 2 | 1 | 13 |
| Koch, 2011^57^ | 2 | 2 | 2 | 1 | 0 | 2 | 2 | 1 | 12 |
| Giacomini, 2010^58^ | 2 | 1 | 2 | 2 | 2 | 2 | 2 | 1 | 14 |
| Jang, 2009^59^ | 2 | 1 | 1 | 2 | 2 | 2 | 2 | 1 | 13 |
| Most, 2006^60^ | 2 | 2 | 2 | 2 | 0 | 2 | 2 | 1 | 13 |
| Sedwick, 2005^5^ | 2 | 2 | 1 | 2 | 0 | 2 | 0 | 0 | 9 |
| Dyer, 2000^62^ | 2 | 1 | 2 | 2 | 0 | 2 | 2 | 1 | 12 |
| Murrell, 2000^63^ | 2 | 1 | 0 | 2 | 0 | 2 | 0 | 0 | 7 |
| Kamami, 2000^64^ | 0 | 1 | 1 | 2 | 2 | 2 | 2 | 0 | 10 |
| Kamami, 1997^65^ | 2 | 2 | 1 | 2 | 1 | 2 | 2 | 1 | 13 |
| Metzinger, 1994^66^ | 2 | 1 | 2 | 2 | 1 | 2 | 2 | 0 | 12 |
