# Supplementary material for: Current Practices and Evidence in Caudal Septoplasty: A National Survey and Systematic Review
Source: Aesthet Surg J Open Forum. 2025 Dec 19;8:ojaf170. doi: 10.1093/asjof/ojaf170 (PMC12862218; doi:10.1093/asjof/ojaf170)
Supplement: ojaf170_Supplementary_Data [file ojaf170_supplementary_data.zip › Supplemental Table 3.docx]

**Supplemental Table 3.** MINORS Assessment Tool for Non-randomized Comparative Studies (n = 8)

| Item | A clearly stated aim | Inclusion of consecutive patients | Prospective collection of data | Endpoints appropriate to the aim of the study | Unbiased assessment of the study endpoint | Follow-up period appropriate to the aim of the study | Loss to follow-up less than 5% | Prospective calculation of the study size | An adequate control group | Contemporary groups | Baseline equivalence of groups | Adequate statistical analyses | Total score |
| --- | --- | --- | --- | --- | --- | --- | --- | --- | --- | --- | --- | --- | --- |
| Ghosh, 2024^16^ | 2 | 2 | 2 | 2 | 2 | 2 | 2 | 2 | 2 | 2 | 2 | 2 | 24 |
| Awan, 2021^27^ | 2 | 1 | 2 | 2 | 2 | 2 | 2 | 2 | 2 | 2 | 2 | 2 | 23 |
| Lip Ng, 2019^33^ | 1 | 2 | 1 | 2 | 1 | 0 | 1 | 2 | 1 | 1 | 1 | 1 | 14 |
| Sazgar, 2019 ^34^ | 2 | 1 | 2 | 1 | 0 | 2 | 2 | 2 | 2 | 2 | 1 | 0 | 17 |
| Karadavut, 2016^46^ | 2 | 2 | 2 | 2 | 2 | 2 | 2 | 1 | 2 | 2 | 2 | 2 | 23 |
| Kayabasoglu, 2015^47^ | 2 | 2 | 2 | 2 | 2 | 2 | 2 | 2 | 1 | 2 | 2 | 2 | 23 |
| Kim, 2011^55^ | 2 | 2 | 2 | 2 | 1 | 2 | 2 | 1 | 1 | 1 | 0 | 2 | 18 |
| Calderón-Cuéllar, 2003^61^ | 2 | 2 | 2 | 2 | 2 | 2 | 2 | 2 | 2 | 2 | 2 | 2 | 24 |
